# Genome-wide identification of novel flagellar motility genes in *Pseudomonas syringae* pv. *tomato* DC3000

**DOI:** 10.3389/fmicb.2025.1535114

**Published:** 2025-01-28

**Authors:** Zichu Yang, Tyler Helmann, Maël Baudin, Karl J. Schreiber, Zhongmeng Bao, Paul Stodghill, Adam Deutschbauer, Jennifer D. Lewis, Bryan Swingle

**Affiliations:** ^1^Plant Pathology and Plant-Microbe Biology Section, School of Integrative Plant Science, Cornell University, Ithaca, NY, United States; ^2^Emerging Pests and Pathogens Research Unit, Robert W. Holley Center, United States Department of Agriculture-Agricultural Research Service, Ithaca, NY, United States; ^3^Department of Plant and Microbial Biology, University of California, Berkeley, Berkeley, CA, United States; ^4^Plant Gene Expression Center, United States Department of Agriculture-Agricultural Research Service, Berkeley, CA, United States; ^5^Institut Agro, INRAE, IRHS, SFR QUASAV, Université Angers, Angers, France; ^6^Environmental Genomics and Systems Biology Division, Lawrence Berkeley National Laboratory, Berkeley, CA, United States

**Keywords:** *Pseudomonas syringae*, flagella, swimming motility, chemotaxis, Tn-seq, essential genes

## Abstract

*Pseudomonas syringae* pv. *tomato* DC3000 (*Pst* DC3000) is a plant pathogenic bacterium that possesses complicated motility regulation pathways including a typical chemotaxis system. A significant portion of our understanding about the genes functioning in *Pst* DC3000 motility is based on comparison to other bacteria. This leaves uncertainty about whether gene functions are conserved, especially since specific regulatory modules can have opposite functions in sets of *Pseudomonas*. In this study, we used a competitive selection to enrich for mutants with altered swimming motility and used random barcode transposon-site sequencing (RB-TnSeq) to identify genes with significant roles in swimming motility. Besides many of the known or predicted chemotaxis and motility genes, our method identified PSPTO_0406 (*dipA*), PSPTO_1042 (*chrR*) and PSPTO_4229 (hypothetical protein) as novel motility regulators. PSPTO_0406 is a homolog of *dipA*, a known cyclic di-GMP degrading enzyme in *P. aeruginosa*. PSPTO_1042 is part of an extracytoplasmic sensing system that controls gene expression in response to reactive oxygen species, suggesting that PSPTO_1042 may function as part of a mechanism that enables *Pst* DC3000 to alter motility when encountering oxidative stressors. PSPTO_4229 encodes a protein containing an HD-related output domain (HDOD), but with no previously identified functions. We found that deletion and overexpression of PSPTO_4229 both reduce swimming motility, suggesting that its function is sensitive to expression level. We used the overexpression phenotype to screen for nonsense and missense mutants of PSPTO_4229 that no longer reduce swimming motility and found a pair of conserved arginine residues that are necessary for motility suppression. Together these results provide a global perspective on regulatory and structural genes controlling flagellar motility in *Pst* DC3000.

## Introduction

Diverse microbial habitats have facilitated evolution of equally diverse specialized molecular systems or modification upon existing structures to enable adaptive behaviors. Bacterial flagella are highly conserved structures that power active motility on surfaces, semi-solid and fluid habitats, and help fulfill ecological functions including accessing nutrients, pathogenesis, social interactions and biofilm formation (Limoli et al., 2019; Bao et al., 2020; Wang et al., 2020; Colin et al., 2021; Wadhwa and Berg, 2022). The flagellum and chemotaxis regulation are the foundation of swimming and swarming motility that has been well-characterized in model systems like *E. coli* (Berg and Anderson, 1973; Wadhwa and Berg, 2022). However, other pathways of flagellar motility regulation have only recently started to be systematically examined and remain understudied (Jones and Elliot, 2017; Baltrus et al., 2018; Pérez-Mendoza et al., 2019; Bao et al., 2020; Yarrington et al., 2024).

*Pseudomonas syringae* pv. *tomato* DC3000 (*Pst* DC3000) is a model plant-pathogenic gram-negative bacterium (Buell et al., 2003). *Pst* DC3000, like its close relative *P. aeruginosa,* has between one and five polar flagella (Sampedro et al., 2015; Munar-Palmer et al., 2024) and appears to share a four-tiered hierarchal transcriptional regulation system that controls production of flagella (Dasgupta et al., 2003). *Pst* DC3000 possesses a typical chemotaxis system that senses the chemical environment by ligand binding and transduces the signal via kinase phosphorylation to modulate flagellar rotation and its directional movement (Sampedro et al., 2015; Clarke et al., 2016). This system includes ligand-binding sensors known as methyl-accepting chemotaxis proteins (MCPs), a methylation-based sensor memory module (CheR and CheB), a kinase-based signal transduction module (CheA, CheY, and CheZ) and structural connection proteins scaffolding the cell pole sensory array (CheV and CheW) (Sampedro et al., 2015; Cassidy et al., 2020). *Pst* DC3000 possesses three *che* clusters (*che1*, *che2*, and *che3*) that each encode seemingly redundant copies of the genes necessary for chemotaxis. However, only *che2* appears to be dedicated to controlling swimming motility (Clarke et al., 2016). *Pst* DC3000 motility regulation has also adapted to serve its specific pathogenic lifestyle, using flagellar motility to invade plant tissues (Nogales et al., 2015; Usuki et al., 2024) but then reducing flagellar expression after entering to avoid activating plant immunity (Bao et al., 2020).

Predicting genes that have motility functions is commonly carried using homology and comparative genomics, but this limits our understanding to only those genes already known to be involved with motility. Identifying novel motility genes requires experimental approaches that do not rely on existing knowledge. TnSeq (transposon site sequencing) is a well-established technique for quantifying each non-essential gene’s contribution to growth in a specified environmental condition (van Opijnen et al., 2009). This technology enables massively paralleled screening of large transposon insertion mutant libraries, with the output being fitness scores calculated by comparing the relative abundance of individual mutants carrying transposon insertions in each gene before and after competitive selection. We used a TnSeq method further optimized for scalability known as random barcode transposon-site sequencing (RB-TnSeq), where each transposon insertion contains a unique barcode sequence that facilitates subsequent quantification of transposon insertion abundance (Wetmore et al., 2015).

In this study, we used RB-TnSeq to assess the contribution of non-essential *Pst* DC3000 genes to motility in a soft agar medium, similar to a previous study in *E. coli* (Girgis et al., 2007). We built and characterized a random barcoded transposon insertion mutant library in *Pst* DC3000 and passaged it on soft agar medium to select for mutants with altered motility (Yu and Alam, 1997; Sampedro et al., 2015). We identified over 70% of known flagellar synthesis and regulatory genes by their fitness phenotypes. Additionally, we focused on three genes of interest: PSPTO_0406 (*dipA*), PSPTO_1042 (*chrR*) and PSPTO_4229 (hypothetical protein) with strong or previously unknown contributions to swimming motility. These results provide a resource for future studies, including a catalog of genes that contribute to *Pst* DC3000 growth and swimming motility and empirical evidence for motility genes previously annotated by orthology.

## Materials and methods

### Bacterial strains and growth conditions

Strains used in this study are described in Table 1. *Pst* DC3000 strains were grown at 28°C in King’s B (KB) medium (King et al., 1954) and on KB medium solidified with 1.5% (w/v) agar. *Escherichia coli* TOP10 (Invitrogen) strains were grown at 37°C in LB medium and on LB medium solidified with 1.5% (w/v) agar (Bertani, 1951). Kanamycin was supplied at 50 μg/mL and gentamycin was supplied at 10 μg/mL. Plasmid DNA was isolated using Qiagen Miniprep Kit (Qiagen) from overnight cultured *E. coli* TOP10 cells and subsequently used to transform *Pst* DC3000 and mutant derivatives by electroporation (Choi et al., 2006).

**Table 1:** Strains and plasmids used.

Strain or plasmid	Relevant characteristic(s)	References
PS1	*P. syringae* pv. *tomato* DC3000 wild type	Buell et al. (2003)
PS129	*P. syringae* pv. *tomat*o DC3000 wild type with pBS60 control plasmid	Markel et al. (2016)
PS398	*P. syringae* pv. *tomato* DC3000 Δ*cheA2*	Clarke et al. (2016)
PS1433	*P. syringae* pv. *tomat*o DC3000 wild type with pIY25	This work
PS1213	*P. syringae* pv. *tomato* DC3000 ΔPSPTO_0406 (*dipA*)	This work
PS1543	*P. syringae* pv. *tomato* DC3000 ΔPSPTO_1042 (*chrR*)	This work
PS1493	*P. syringae* pv. *tomato* DC3000 ΔPSPTO_4229	This work
PS1450	*P. syringae* pv. *tomato* DC3000 Δ*dipA* with pIY23	This work
PS1451	*P. syringae* pv. *tomato* DC3000 Δ*dipA* with pBS60	This work
PS1546	*P. syringae* pv. *tomato* DC3000 Δ*chrR* with pIY39	This work
PS1547	*P. syringae* pv. *tomato* DC3000 Δ*chrR* with pBS60	This work
PS1495	*P. syringae* pv. *tomato* DC3000 ΔPSPTO_4229 with pIY30	This work
PS1496	*P. syringae* pv. *tomato* DC3000 ΔPSPTO_4229 with pBS60	This work
PS1542	*P. syringae* pv. *tomato* DC3000 ΔPSPTO_1980 (*cheY*)	This work
PS1542	*P. syringae* pv. *tomato* DC3000 Δ*cheY* with pIY38	This work
PS1542	*P. syringae* pv. *tomato* DC3000 Δ*cheY* with pBS60	This work
**Plasmids**		
pBS46	pBBR1MCS5 derivative, Gateway compatible broad-host range expression vector with nptII promoter, Gm^r^	Wei et al. (2007)
pBS60	P_nptII_::empty control vector for pBS46 based expression, Gm^r^	Swingle et al. (2008)
pK18mobsacB	Allelic exchange suicide vector; lacZ mob sacB; Km^r^ Suc^s^	Swingle et al. (2008)
pZB72	pK18MobSacB based PSPTO_0406 (*dipA*) deletion construct, Km^r^	This work
pIY33	pK18MobSacB based PSPTO_1980 (*cheY*) deletion construct, Km^r^	This work
pIY32	pK18MobSacB based PSPTO_1042 (*chrR*) deletion construct, Km^r^	This work
pIY26	pK18MobSacB based PSPTO_4229 deletion construct, Km^r^	This work
pIY23	pBS46 based nptII::*dipA* expression construct, Gm^r^	This work
pIY39	pBS46 based nptII::*chrR* expression construct, Gm^r^	This work
pIY30	pBS46 based nptII::PSPTO*_*4229 expression construct, Gm^r^	This work
pIY38	pBS46 based nptII::*cheY* expression construct, Gm^r^	This work
pIY59	pBS46 based nptII::PSPTO*_*4229 (R84A R85A) expression construct, Gm^r^	This work
pIY25	pBS46 based nptII::*wspR19* expression construct, Gm^r^	This work
**Strain libraries**		
	*E. coli* barcoded Mariner plasmid library	Wetmore et al. (2015)
	*Pst* DC3000 barcoded Mariner plasmid library	This work

### Barcoded transposon library construction

The *Pst* DC3000 random barcoded-transposon insertion library was constructed by conjugating the *E. coli* APA752 donor library containing the barcoded mariner plasmid pKMW3 into *Pst* DC3000. Mating was performed overnight at a 1 (*E. coli*) to 4 (*Pst* DC3000) ratio on nutrient agar (2 g l^−1^ yeast extract, 5 g l^−1^ peptone, 5 g l^−1^ NaCl containing 300 μM diaminopimelic acid). The bacterial mixture was harvested into a single sample, resuspended in liquid KB, plated on KB supplemented with 1.5% (w/v) agar, 50 μg ml^−1^ kanamycin and 50 μg ml^−1^ rifampicin plates and incubated for 2 d at 28°C to select for insertion mutants. Approximately 214,000 mutant clones were obtained, each one representing one transposition event. These clones were pooled together in 200 mL of KB media with 50 μg ml^−1^ kanamycin and 50 μg ml^−1^ rifampicin and 20% glycerol, aliquoted in 1 mL samples and frozen at −80°C.

### Transposon library mapping and essential gene predictions

Genomic DNA from the *Pst* DC3000 library was prepared for sequencing as previously described (Wetmore et al., 2015; Price et al., 2018). In brief, genomic DNA was fragmented by ultrasonication to an average size of 300 bp and an Illumina library preparation was performed based on modifications of the NEBNext DNA library preparation kit (New England Biolabs) with PCR enrichment of the transposon insertion sites. Mapping and pool construction were performed using the FEBA scripts MapTnSeq.pl. and DesignRandomPool.pl.,^1^ requiring a minimum of 10 reads per barcode to be included in the mapping (Wetmore et al., 2015). Based on the TnSeq mapping data, we used feba/bin/Essentiality.pl. as in Price et al. (2018) to calculate the read density (reads/nucleotides across the entire gene) as well as the insertion density (sites/nucleotides) within the central 10–90% of each gene. We then used the “Essentials” function in feba/bin/comb.R (Price et al., 2018), to predict likely essential genes. This analysis estimates how short a gene could be and still be unlikely to have no insertions by chance (*p* < 0.02, Poisson distribution). After excluding genes shorter than 350 bp, the read density was normalized by GC content, and the insertion density was normalized so the median gene’s value was 1. Genes were considered essential or nearly essential if both the normalized read density and the normalized insertion density were < 0.2. Because mariner transposons require a TA dinucleotide site for insertion, genes with 0 TA sites were also excluded.

### *In vitro* fitness assays

One aliquot (1 mL) of the library glycerol stock was thawed and resuspended to an OD_600_ of approximately 0.3 in 20 mL of KB media supplemented with 50 ug/mL rifampicin and 50 ug/mL kanamycin. The culture was incubated at 30°C until the OD_600_ had doubled, at which time an aliquot was harvested to serve as the time 0 (T0) sample. The remaining cells were pelleted, washed twice with M9 minimal media, and resuspended to a final OD_600_ of 0.02 in 5 mL of M9 minimal media ± 2 mg/mL Casamino acids. Three replicates of each culture condition were incubated at 30C until they achieved an OD_600_ of approximately 1 (close to six doublings), then harvested for BarSeq.

### Soft agar swimming assays

MOPS minimal media supplied with 0.2% fructose and 0.3% agar was used for all swimming assays (Neidhardt et al., 1974). Five milliliter of swimming assay medium was added to 35 × 10 mm petri dish and air dried in a biosafety cabinet hood for exactly 30 min before inoculation. *Pst* DC3000 strains overnight cultures were resuspended and adjusted to an OD_600_ of 0.4 in MOPS liquid media with 0.2% fructose prior to inoculation. One microliter inoculum from *Pst* DC3000 wild type or derivative strain culture was injected into the agar at the geometrical center of the petri dish. Plates were then transferred to a sealed moisture-enhanced chamber (RH = 100%) and incubated at 25°C for 48 h. For the batch swimming assay, 50 mL of swimming assay medium was added to square plates (86 × 128 mm) instead.

### Passaging between consecutive series of liquid culture and soft swimming assays

**Figure 1:**
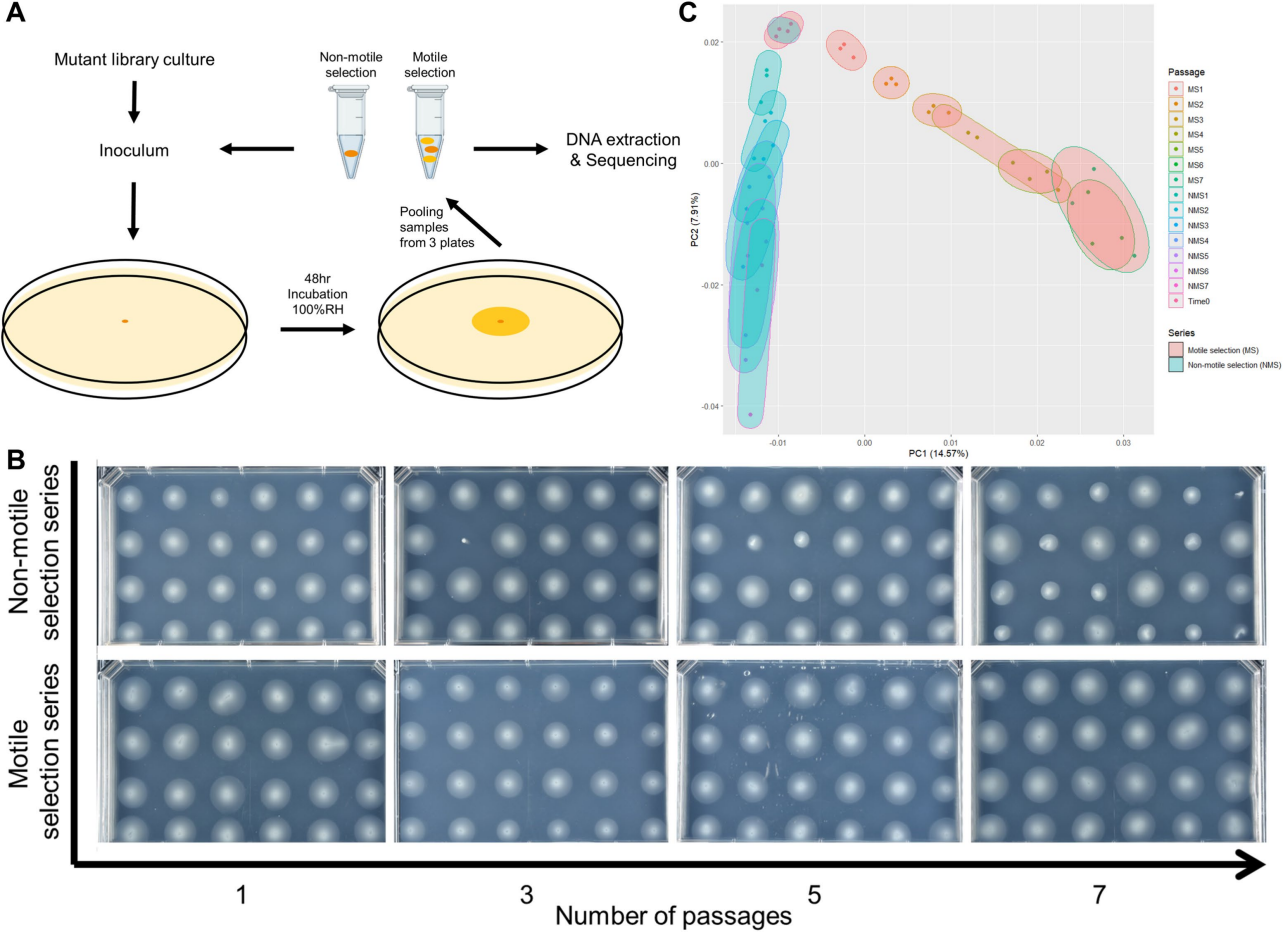
Selection for reduced motility led to population-wide phenotypic and genetic shift of *Pst* DC3000 barcoded transposon mutant library composition. **(A)** Graphic explanation of serial swimming assay passaging experiments. Pooled strains from the mutant library were used as initial inoculum. In each passage, cells from the whole swimming colony (motile selection series, or MS) or those that failed to move from the inoculation point after 48 h (non-motile selection series, or NMS) were collected and a fraction was used to inoculate fresh swimming medium. The remaining samples were processed for BarSeq analysis to measure strain abundance. **(B)** Population-wide shift in swimming motility phenotype across 7 passages of serial swimming assays. Single colonies were isolated from the samples taken after each passage and phenotyped for swimming motility. Passaging populations in the serial swimming assays with selection for low motility enriched for mutants with impaired swimming motility. **(C)** Principal component analysis (PCA) of gene fitness scores from all 18 samples collected at the end of each passage and time 0 samples. Selection for low motility in serial swimming passages caused strong shift in the genomic profiles of fitness scores.

Initial inoculum generated from the RB-TnSeq library was prepared similarly as described above but from a recovery culture where a library aliquot was thawed on ice and added to 50 mL of LB with 50 μg/mL kanamycin. This recovery culture was then incubated at 28°C for 6 h before inoculum preparation. For liquid culture passages, a 1 μL inoculum from the library recovery culture was added to 5 mL of MOPS minimal media with 0.2% fructose and grown for 48 h at 25°C with shaking. At 48 hpi, this culture was spun down and cells were extracted for making new inoculum. Motility assays in both motile and non-motile selection series were set up as described above. For the motile selection series, the whole swimming colony was extracted from the passaging swimming plates with a 6 mm radius biopsy punch (Robins Instruments) at 48 hpi while a sample of cells was extracted from the inoculation point by a 200 μL micropipette for the non-motile selection series (Figure 1A). Bacterial cells were then released from the soft agar by physical homogenizing and resuspended into novel inoculum at OD_600_ of 0.4 in MOPS liquid media with 0.2% fructose.

### Library pre-culture, BarSeq PCR, Illumina sequencing and gene fitness calculations

For the library pre-culture, BarSeq PCR, Illumina sequencing and fitness calculations, the exact protocol was followed as previously described (Helmann et al., 2019). Genomic DNA was purified using Wizard SV Genomic DNA Purification System (Promega), and the 98°C BarSeq PCR as described by Wetmore and colleagues was used to amplify the barcode region of each sample (Wetmore et al., 2015). The PCR for each sample was performed in 50 μL total volume: containing 0.5 μL Q5 High-Fidelity DNA polymerase (New England Biolabs, United States), 10 μL 5X Q5 buffer, 10 μL 5X GC enhancer, 1 μL 10 mM dNTPs, 150–200 ng template gDNA, 2.5 μL common reverse primer (BarSeq_P1), and 2.5 μL of forward primer from one of the 96 indexed forward primers (BarSeq_P2_ITXXX), both at 10 μM (Wetmore et al., 2015). Following the BarSeq PCR, 10 μL of each reaction was pooled, and 200 μL of this DNA pool was subsampled and purified using the DNA Clean and Concentrator Kit (Zymo Research, United States). The final DNA sequencing library was eluted in 30 μL nuclease-free water, quantified on a Nanodrop One (ThermoFisher, United States), and submitted for sequencing at the BRC Genomics Facility at the Cornell Institute of Biotechnology. Prior to sequencing, the quality of each amplicon pool was assessed using a Bioanalyzer. Each sequencing pool was run on a single NextSeq 500 (Illumina, Inc., United States) lane for 75 bp single-end reads.

Sequencing reads were used to calculate genome-wide gene fitness using the FEBA scripts MultiCodes.pl., combineBarSeq.pl., and BarSeqR.pl. (Wetmore et al., 2015). From the raw fastq reads for each sample, individual barcode sequences were identified and counted using MultiCodes.pl., and these counts were combined across samples using combineBarSeq.pl. Using BarSeqR.pl., fitness values for each insertion strain were calculated as the log_2_ ratio of barcode abundance following library growth under a given experimental condition divided by the abundance in the time0 sample. The fitness of each gene is the weighted average of strain fitness values based on the “central” transposon insertions only, i.e., those within the central 10–90% of a gene. Barcode counts were summed between replicate time0 samples. For analysis, genes were required to have at least 30 reads per gene in the time0 sample, and 3 reads per individual strain, and gene fitness values were normalized across the chromosome so that the median gene fitness value was 0 (Wetmore et al., 2015).

### Fitness analysis and plotting

Both fitness scores and an absolute t-like statistic were used to prioritize genes of interest. This *t*-value was calculated as the fitness score divided by the square root of the maximum variance calculated in two ways (Wetmore et al., 2015). High fitness value genes were selected by criteria fitness score >2 or <−2 and absolute t-score > 5. Data analysis was conducted in R v.4.2.3 and data visualization were carried out with ggplot2. Principal component analysis was performed on the gene fitness matrix across all 14 passages excluding time0 with R package prcomp.

### Cloning of *Pst* DC3000 mutants and complemented strains

Standard marker exchange mutagenesis was used to sequentially produce a *Pst* DC3000 strain with each gene of interest deleted (Kvitko and Collmer, 2011). Deletion constructs for PSPTO_1042 (pIY32) and PSPTO_1980 (pIY33) were each made using synthetic linear DNA fragments from Twist Bioscience that contained between 800 to 1,000 bp flanking both ends of each gene joined together with *Eco*RI and *Hind*III sites added to the 5′ and 3’ends, respectively (Supplementary Table S5). Each deletion construct retained the first and last six codons of the original gene to minimize polar effects on downstream genes. The DNA fragments were incorporated into pK18MobSacB by restriction enzyme digestion and ligation with T4-ligase (Thermo Fisher Scientific). Deletion construct for PSPTO_0406 pZB72 was created by PCR amplification of 0.7 and 0.8-kb flanks to PSPTO_0406 with oSWC4709/4711 and oSWC4712/4713, respectively. Then, gel purified PCR fragments were joined by a second PCR amplification with primers oSWC4710/4714 containing XbaI restriction sites. The approximately 1.4 kb product was gel purified, digested with XbaI and ligated with pK18mobsacB cut with the same restriction enzyme. The deletion construct for PSPTO_4229 (pIY26) was created in a similar fashion. Deletion constructs were confirmed by restriction analysis and sequencing before transformed into wild-type *Pst* DC3000 by electroporation (Choi et al., 2006) and selection for kanamycin resistant merodiploids. Sucrose was used to select for recombinants that had subsequently eliminated the *sacB*-containing deletion construct plasmid backbone and confirmed to have lost kanamycin resistance. The deletion was confirmed by sequencing PCR products amplified with primers that anneal to sequences flanking the deleted locus.

Overexpression plasmids were constructed with Gateway cloning. An expression cassette of each target gene was PCR amplified with CACC motif on 5′ end and incorporated in pENTR/D/SD backbones using TOPO cloning (Thermo Fisher Scientific). Then these DNA fragments were incorporated into pBS46 (Swingle et al., 2008) with Gateway LR Clonase II enzyme mix (Thermo Fisher Scientific). After whole plasmid sequencing (Plasmidsaurus) confirmation, these plasmids were then used to transform corresponding *Pst* DC3000 deletion mutant strains by electroporation (Choi et al., 2006). The transformants were then selected by Gentamycin resistance.

## Results

### Creation of barcoded transposon libraries in *Pst* DC3000 and prediction of essential genes

To screen for *Pst* DC3000 genes important in swimming motility, we constructed a library of random barcoded insertion mutants using a barcoded mariner transposon (Wetmore et al., 2015). High-throughput sequencing was used to map insertion locations in the genome as well as their corresponding 20 nucleotide barcode sequences. We identified 125,581 barcodes that were seen 10 or more times and mapped to the genome at 73,967 different locations. Of these, 73,138 insertions (at 43,173 distinct locations) were located in the central 10–90% of coding sequences and could therefore be used for fitness analysis (4,691 of 5,619 protein-coding genes). Overall, this mutant library consists of a median of 11 insertion strains per protein-coding gene. We also used these mapping data to predict 403 (8%) putative essential genes on the chromosome, based on protein-coding genes containing at least one TA dinucleotide site and at least 350 bp in length, while having no or very low abundance of transposon insertions (Supplementary Table S1). Enriched functional category annotations among these genes include translation, gene expression, cell wall biogenesis, lipid biogenesis and cofactor metabolic process (Supplementary Table S1).

### Serial passaging enrichment experimental design and derivation of fitness scores

Swimming in soft agar media is generally adaptive because it helps cells access nutrients and reduce competition with other clonemates. We predict that when a colony develops from a library of pooled mutants, motile cells will move from the inoculation point toward the periphery and leave the less motile mutants enriched in the center of the colony. Sampling the diversity in an entire colony after it forms should show a bias for fully motile mutants and a depletion of non-motile mutants. This bias should be reversed in the center of the colony near the inoculation point, where there should be an enrichment for non-motile and less-motile mutants. In contrast, growth in liquid with shaking moves the bacteria by external forces and should reduce or eliminate the adaptive benefits that flagella provide, while still maintaining selection for genes needed for growth.

We passaged our barcoded transposon insertion mutant library in swimming medium and sampled the entire colony or only the center at the inoculation point to select for the motile and non-motile populations, respectively. As a baseline control, we grew the library in liquid media to identify genes needed for growth and survival in the medium. In all cases we used MOPs minimal medium with 0.2% fructose (m/v) that was either prepared as liquid or semi-solidified with 0.3% agar (m/v) (Neidhardt et al., 1974). All three selection series were started from the same culture of the barcoded transposon insertion mutant library (Time 0 sample) and replicated in triplicate. For the liquid selection series, we inoculated MOPs liquid medium and incubated for 48 h at 25°C with shaking before sampling for DNA extraction and to inoculate a second passage. For the motile and non-motile selection series, the inoculum was injected into the medium and the colony was allowed to grow for 48 h at 25°C in a humid chamber. We then collected the cells either from the entire colony (motile selection series) or only from the area in the vicinity of the inoculation point (non-motile selection series) (Figure 1A and Methods). Cells recovered from the motile and non-motile selection series were then each used for DNA extraction and as inocula for subsequent passages in swimming medium. This isolation and re-inoculation cycle was repeated for a total of 7 passages for the motile and non-motile selection series.

We followed the formula developed by Wetmore and colleagues for calculating fitness scores (Wetmore et al., 2015). Mutant fitness scores were calculated as the weighted average of the log2 of the ratio of barcode abundance after each passage to that of barcode abundance in the time 0 samples, then the gene fitness scores as the weighted average of fitness scores for all strains with transposon insertions in each gene (Wetmore et al., 2015). Since fitness scores are calculated based on the abundance of typically loss-of-function mutants, positive-value fitness scores are associated with genes whose function are antagonistic for fitness in the specified environment and negative fitness scores are conversely associated with genes that contribute a beneficial function under the imposed selection. We focused our analysis on genes with absolute fitness score values above 1 and considered genes with absolute fitness score values above 2 and absolute t-scores above 5 to have strong influence on fitness and survival. We found 30 genes from the liquid series, 202 genes from the motile selection series and 153 genes from the non-motile selection series with absolute fitness scores above the strong influence threshold (i.e., fitness score absolute values of greater than 2) (Table 2; Supplementary Table S2). We conducted Gene Ontology (GO) annotation enrichment analysis on the 30 genes identified in the liquid series and as expected found amino acid metabolism and organic acid biosynthesis to be the most strongly enriched functional categories (Supplementary Table S2), suggesting these genes are necessary for growth under the nutritional restriction of MOPs minimal liquid media.

**Table 2:** Motility-altering gene candidates identified by PCA thresholding.

Series	Total	# of genes with positive fitness scores	# of genes with negative fitness scores
Liquid	30	0	30
Motile selection	202	52	150
Non-motile selection	153	44	109

We assessed the efficacy of the non-motile selection for mutants with defects in motility by testing the swimming phenotype of randomly sampled individual strains from the populations collected after each passage. For comparison, we determined the swimming phenotypes of individuals sampled from the motile selection series passages where the imposed selection favored motile cells. The motile selection series showed little to no change in motility across the seven passages (Figure 1B). In contrast, the proportion of mutants with motility defects steadily increased in non-motile selection series (Figure 1B). The effect of selection for low motility was also evident in the landscape of gene fitness scores where a clear divergence emerged in the principal component analysis between the non-motile selection series compared to the motile selection series (Figure 1C).

### Motility is strongly beneficial for *Pst* DC3000 growth in swimming medium

**Figure 2:**
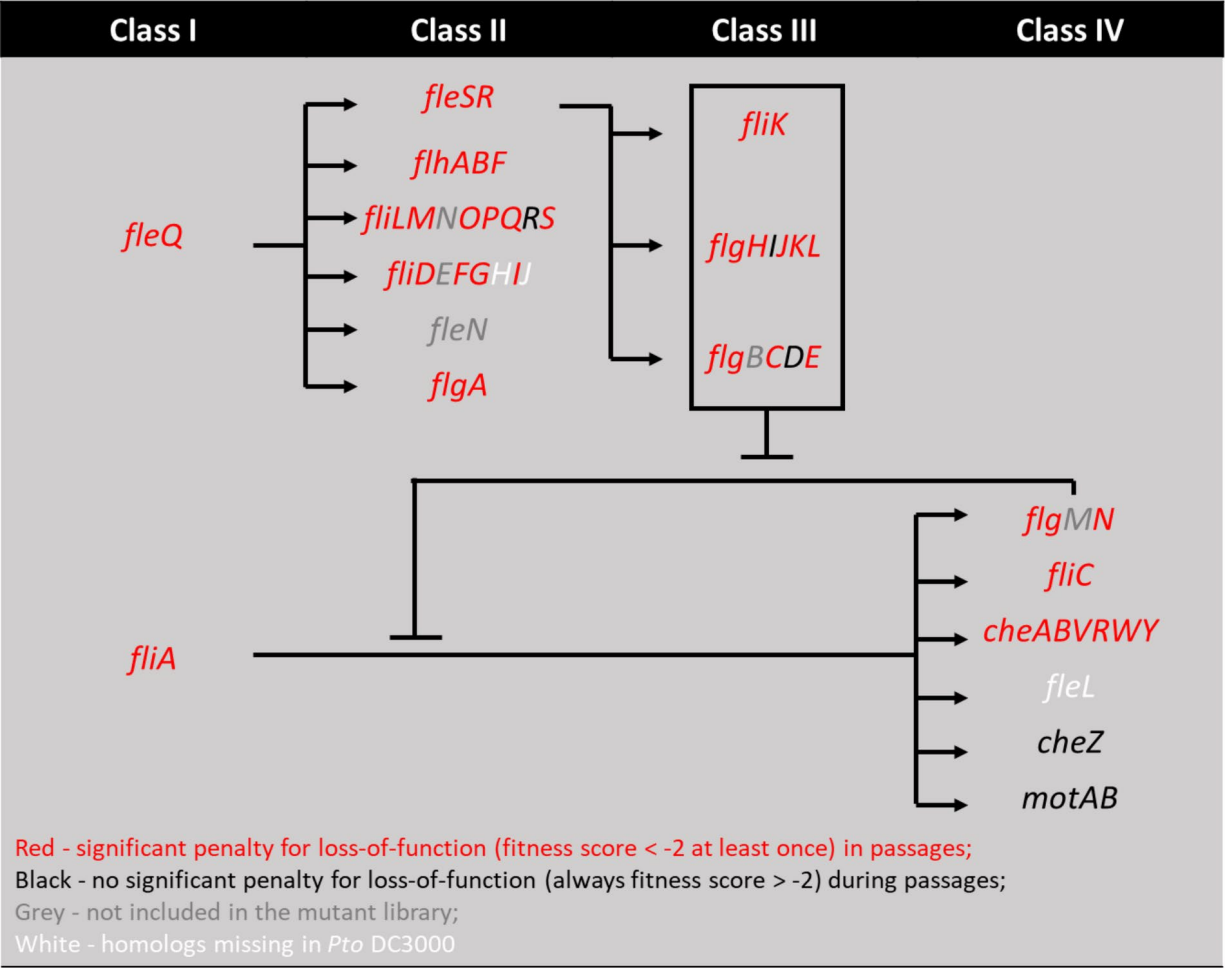
Mapping genes with high fitness scores in motile selection series onto the four-classes flagellar motility regulation pathways from *P. aeruginosa* model (Dasgupta et al., 2003). Genes with fitness score below −2 at any time during the 7 passages in motile series are marked red and otherwise black. Genes present in *Pseudomonas aeruginosa* but not in *Pst* DC3000 are marked white. Genes present in *Pst* DC3000 but filtered out by the analysis pipeline or not included in the mutant library are marked grey. We note that while *fliR* and *flgI* are marked black for fitness scores below threshold, *flgD*, *cheZ*, and *motAB* are marked black for their above threshold (absolute value > 2) positive fitness scores, suggesting they function to inhibit motility.

We found 202 genes that strongly influenced fitness and survival in the motile selection series (absolute values above 2). We compared this list of genes to those found in the liquid series control to help categorize the roles of genes in the motile selection series. We found that 29 of the 30 genes identified with high fitness scores (absolute values above 2) in the liquid series also had similar high-absolute-value scores in the motile selection series, indicating that the nutritional requirements are the same in both conditions. We examined the GO annotation of the remaining 173 genes and found motility was the most strongly enriched category of annotations (Fisher’s exact test, FDR-adjusted *p* < 0.001, Supplementary Table S2). Consistent with our hypothesis that motility is beneficial for growth in soft agar swimming media, we found homologs of 34 out of 47 genes in the *P. aeruginosa* flagellar synthesis and regulatory pathways (Dasgupta et al., 2003) showed strongly negative fitness scores across the passages (Figure 2; Supplementary Tables S2, S3), with 44 out of these 47 genes conserved in *Pst* DC3000. The most surprising exception was that *motA* (PSPTO_4953) and *motB* (PSPTO_4952), which stood out as having strongly positive fitness scores, indicating that these flagellar motor proteins are paradoxically antagonistic for growth in swimming media.

These results suggest that thresholding genetic fitness scores can indeed identify genes with a strong impact on motility. However, results from this thresholding approach do not compare the relative magnitude of each gene’s impact on swimming motility and thus does not help identify the most likely novel motility regulators. We therefore needed to find a quantitative approach to rank genes in their likelihood of encoding motility regulators regardless of their current annotation.

### Difference in gene fitness scores between motile and non-motile selection series predict contributions to motility

Non-motile selection should enrich for and increase the relative fitness of mutants with insertions in genes that contribute positively to motility and cause these genes to have increased fitness scores, in contrast to the motile selection. Consistent with this, we observed that known motility genes like *cheY* (PSPTO_1980) displayed much increased fitness scores in the non-motile selection series compared to the motile selection series. We therefore compared gene fitness scores throughout the non-motile selection series to those throughout the motile selection series, with the expectation that the magnitude of the difference in fitness scores should correlate with the genes’ contribution to motility. We found 600 genes with fitness scores that differed between the two series across passages (t-test, FDR-adjusted *p* < 0.05), and again found chemotaxis and motility as the most enriched functional category (Supplementary Table S4). This approach successfully identified major components of the chemotaxis regulation *cheAYVRW* and 10 MCPs but failed to identify flagellar genes from class I to III (Supplementary Table S4) (Dasgupta et al., 2003).

**Figure 3:**
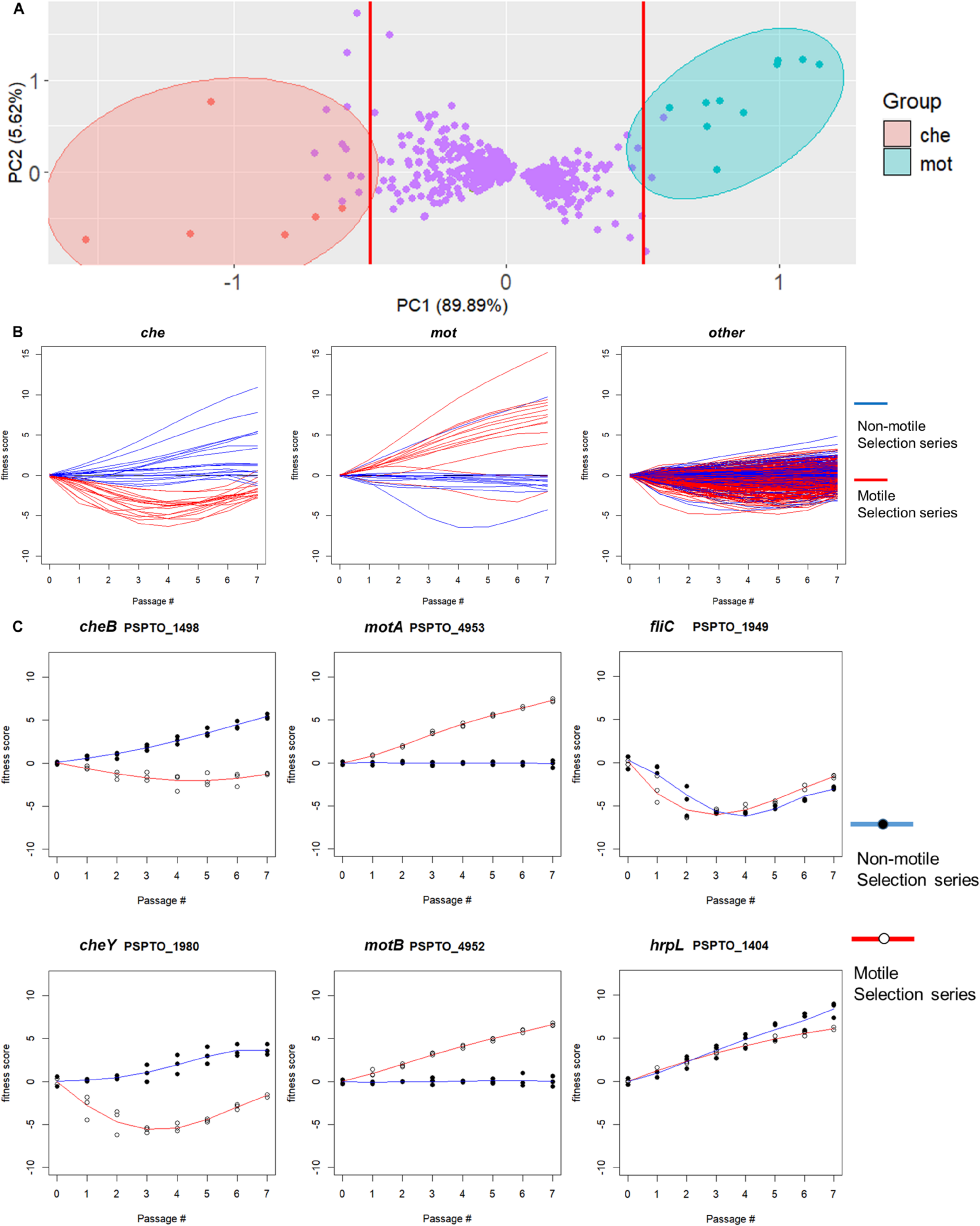
Difference in gene fitness score trends between non-motile and motile selection passages identifies potentially motility-contributing and motility-antagonistic genes. **(A)** Principal component analysis (PCA) of per-passage fitness score differences separates all 600 identified differentially fit genes (DFGs) into three major groups along PC1. A PC1-based threshold at values of −0.5 and 0.5 (red lines) was used to identify two groups of potentially motility-altering genes represented by *cheY* (referred to as *che* genes) and *motAB* (referred to as *mot* genes), respectively. **(B)** Fitness score profiles of representative genes from each DFG group. The *che* group genes (left) showed negative fitness scores in the motile selection series and reversal to positive trend in the non-motile selection series. The *mot* group genes (middle) displayed a positive trend of fitness scores in the motile selection series and shifted to a neutral or slightly negative trend in the non-motile selection series. The remaining genes (right) displayed fitness scores with less divergence between the two series. **(C)** Fitness scores in motile and non-motile selection series for selected representative genes. Chemotaxis regulators *cheY* and *cheB* represent *che* group and *motAB* represent mot group. Flagellin encoding *fliC* and Type 3 secretion system regulator *hrpL* were chosen from the non-DFGs as controls that show no divergence between the two series.

We used the fitness score differences between the two selection series as a quantitative metric for ranking genes according to their likelihood of functioning in motility and conducted principal component analysis (PCA) on the resulting profiles of 600 differentially fitness genes (DFG). We found two groups of motility-altering genes that were strongly separated by PC1, each including known motility-related genes such as chemotaxis response regulator *cheY* and flagellar stator *motAB*, respectively (Figure 3A). Clustering results from K-means analysis (X = 4) similarly identified the two groups of genes containing *cheY* and *motAB*, respectively, as unique clusters (Supplementary Figure S1), suggesting there is functional similarity among genes within each group. We thus used PC1 value of −0.5 and 0.5 as thresholds to identify individual genes in each of the two groups as being potentially important for swimming motility (vertical lines, Figure 3A). We found 18 genes in the *che* group that presumably contribute to swimming motility and 13 genes in the *mot* group that appeared antagonistic to swimming motility (Table 3). Genes in the *che* group displayed negative fitness scores in the motile selection series, with a complete reversal to positive trend in the non-motile selection series (Figure 3B, left panel) while the *mot* group genes displayed a positive trend of fitness scores in the motile selection series and shifted to a neutral, slightly negative trend in the non-motile selection series (Figure 3B, middle panel). Genes from both *che* and *mot* groups displayed stronger divergence in their fitness scores between motile and non-motile series than the rest of DFGs (Figures 3B,C). These results are consistent with our hypothesis that genes affecting swimming motility would have fitness scores that differ most between the two series.

**Table 3:** Motility-altering gene candidates identified by PCA thresholding.

Locus tag	Description	PC1 value	Fitness profile group
PSPTO_0406	Sensory box/GGDEF domain/EAL domain-containing protein	−1.55	*che*
PSPTO_4229	Hypothetical protein	−1.16	*che*
PSPTO_1980	Chemotaxis protein CheY	−1.08	*che*
PSPTO_1042	Transcriptional activator ChrR	−0.81	*che*
PSPTO_1498	Protein-glutamate methylesterase CheB	−0.70	*che*
PSPTO_1727	Hypothetical protein	−0.70	*che*
PSPTO_3821	Lipoprotein	−0.66	*che*
PSPTO_0751	Hypothetical protein	−0.65	*che*
PSPTO_1496	Chemotaxis protein CheW	−0.60	*che*
PSPTO_1755	Glycoside hydrolase family protein	−0.60	*che*
PSPTO_4868	Sensor histidine kinase/response regulator RetS	−0.60	*che*
PSPTO_1982	Chemotaxis sensor histidine kinase CheA	−0.59	*che*
PSPTO_1987	Chemotaxis protein CheW	−0.59	*che*
PSPTO_5230	Flagellar basal body-associated protein FliL-like protein	−0.59	*che*
PSPTO_1911	Response regulator/TPR domain protein	−0.56	*che*
PSPTO_2173	Isopropylmalate isomerase large subunit	−0.55	*che*
PSPTO_1912	Sensor histidine kinase	−0.54	*che*
PSPTO_3910	Hypothetical protein	−0.53	*che*
PSPTO_4478	4-carboxymuconolactone decarboxylase	0.51	*mot*
PSPTO_2275	Phospho-2-dehydro-3-deoxyheptonate aldolase	0.53	*mot*
PSPTO_1935	Basal-body rod modification protein FlgD	0.58	*mot*
PSPTO_2222	Sensor histidine kinase	0.60	*mot*
PSPTO_0116	LysR family transcriptional regulator	0.73	*mot*
PSPTO_4952	Flagellar motor protein MotB	0.73	*mot*
PSPTO_5482	Response regulator	0.77	*mot*
PSPTO_4953	Flagellar motor protein MotA	0.79	*mot*
PSPTO_0413	Hypothetical protein	0.87	*mot*
PSPTO_3229	Filamentous hemagglutinin, intein-containing	0.99	*mot*
PSPTO_1732	Hypothetical protein	1.00	*mot*
PSPTO_3890	FKBP-type peptidyl-prolyl cis-trans isomerase	1.08	*mot*
PSPTO_3230	Hemolysin activator protein, HlyB family	1.15	*mot*

### Characterization of individual motility-contributing genes with strong fitness phenotypes

**Figure 4:**
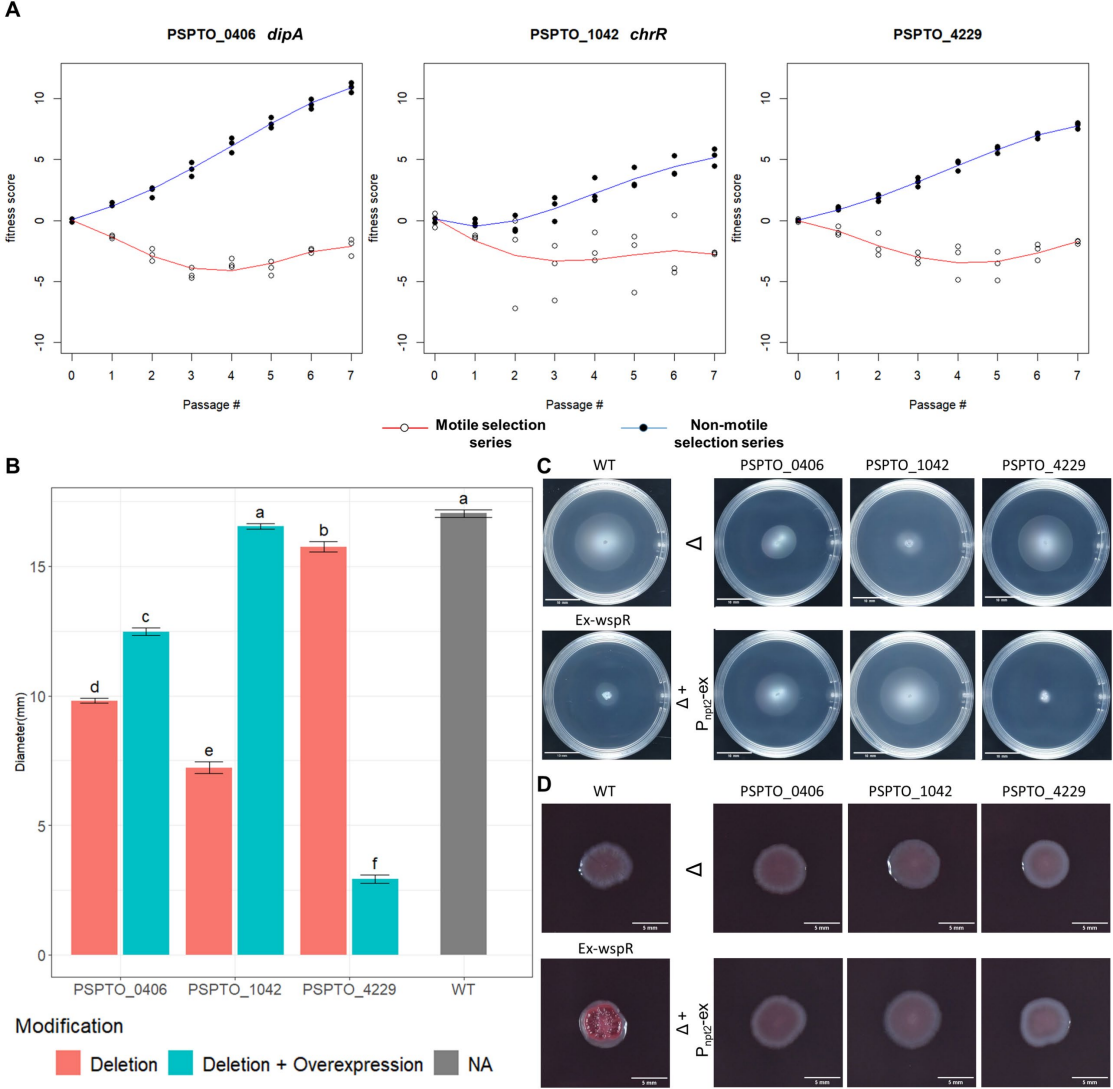
Phenotypic characterization of TnSeq-identified motility regulator genes. **(A)** Fitness score profile of PSPTO_0406 (*dipA*), PSPTO_1042 (*chrR*) and PSPTO_4229 from both non-motile selection series and motile selection series of passages. **(B)** Quantification of colony diameters of each characterized strain at 72hpi in MOPs minimal media swimming assay. Letter group was assigned by post-hoc Tukey test after one-way ANOVA. Error bars represent standard mean of error (SEM). **(C)** Representative swimming colony morphology at 72 h in MOPs minimal media swimming plates. Positive (wildtype) and negative (*wspR* overexpression) control phenotypes are listed on the left. Knockout strains for each gene are represented on the top raw and denoted with *Δ* while the nptII promoter-based complement strain are displayed below the cognate knockout strains and are denoted with Δ+P_npt2_-ex. **(D)** Colony morphology of each strain at 96 hpi in Congo Red assay. (Ex-wspR) overexpression of *P. fluorescens wspR19* in *Pst* DC3000 (WT) as positive control for Congo red binding.

We next investigated individual genes from the predicted motility-contributing *che* group because their mutant phenotypes should be easily identifiable in swimming assays. We chose PSPTO_0406 (*dipA*), PSPTO_1042 (*chrR*) and PSPTO_4229 (hypothetical protein) as our candidate motility-contributing genes because their fitness score differences between non-motile and motile selection series strongly suggest they positively contribute to swimming motility (Figure 4A).

Motility and biofilm formation are inversely controlled by c-di-GMP levels in the cell, with low c-di-GMP levels favoring biofilm dispersal and motility (Christen et al., 2010; Matsuyama et al., 2016; Khan et al., 2023; Kreiling and Thormann, 2023). Cellular c-di-GMP levels are managed by sets of phosphodiesterase (PDEs) and diguanylate cyclase (DGCs) enzymes that degrade and synthesize c-d-GMP, respectively (Khan et al., 2023; Kreiling and Thormann, 2023). Among the genes identified as contributing to motility, PDE-encoding PSPTO_0406 (*dipA*) had the largest response to selection for low motility (Figure 4A). We generated a genomic *dipA* knock-out mutant as well as *dipA* overexpression plasmid to assess its impact on swimming motility. Both *dipA*^+^ overexpression strain and Δ*dipA* mutant were partially impaired swimming motility compared to the wild-type *Pst* DC3000 (Figures 4B,C). The relative levels of c-di-GMP can be compared using Congo red, which binds exopolysaccharide produced while c-di-GMP levels are elevated (Bordi et al., 2010). We grew our strains of interest on Tryptone medium supplied with Congo Red and found neither the *dipA* deletion strain or *dipA*^+^ overexpression strain was morphologically distinguishable from the wild type (Figure 4D) (Bordi et al., 2010). This suggests *dipA* is unlikely to be the dominant PDE in *Pst* DC3000 and that the global c-di-GMP level in wild-type *Pst* DC3000 might already be below detection threshold of the Congo Red assay. Consistent with this result, we saw no difference between these strains using a P*_cdrA_*:gfp reporter (data not shown) (Rybtke et al., 2012; Cerna-Vargas et al., 2019).

PSPTO_1042 (*chrR*) together with PSPTO_1043 encode an ChrR/RpoE-like anti-sigma/sigma factor pair that responds to oxidative stress (Butcher et al., 2017). PSPTO_1043 was proposed to also regulate motility based on its regulation of DGC encoding PSPTO_2591 (Butcher et al., 2017). If true, deletion of PSPTO_1042 *chrR* should de-inhibit PSPTO_1043 activity and lead to strong PSPTO_2591 expression and elevated c-di-GMP level. Deletion of PSPTO_1042 (*chrR*) indeed resulted in strong suppression of swimming motility and the plasmid-based expression fully restored motility level back to wild type (Figures 4B,C). However, we found no visible difference with the Δ*chrR* mutant growing on Congo Red (Figure 4D).

**Figure 5:**
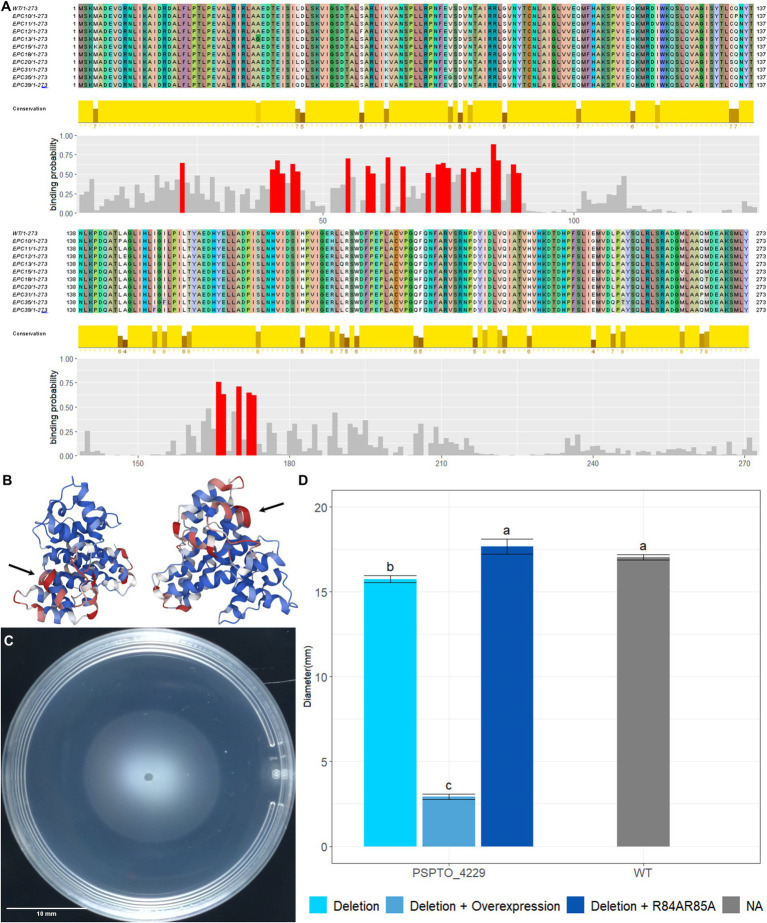
Functional analysis of PSPTO_4229 mutants. **(A)** Alignment of PSPTO_4229 amino acid sequence and full-length mutants produced by error-prone PCR. Coloration of sequence was assigned by Jalview 2.11.3.2 to show amino acids and variation in sequence. Amino acid conservation rates among these sequences are indicated by the height of yellow bars below each site. Protein–protein interaction binding probability of each amino acid was generated by Scannet and positions with >0.5 binding probability are indicated in red. **(B)** Scannet prediction of binding probabilities on AlphaFold2 predicted protein structure of PSPTO_4229. Red coloration indicates high probability of binding. Black arrows point to location of arginine 84 and 85. **(C)** Swimming phenotype of PSPTO_4229 R84A R85A overexpression on plasmid in *Pst* DC3000 PSPTO_4229 deletion mutant. **(D)** Comparison of swimming colony diameters of all tested PSPTO_4229 mutants of *Pst* DC3000 with wild-type *Pst* DC3000 as control. Letter group was assigned by post-hoc Tukey test after one-way ANOVA. Error bars represent SEM.

PSPTO_4229 encodes a hypothetical protein of 273 amino acids with an HD-related output domain (HDOD) (Lee et al., 2016) spanning from amino acids 23 to 210. Deletion of PSPTO_4229 resulted in moderately inhibited swimming motility while over-expression almost abolished swimming completely (Figures 4B,C). Neither deletion nor overexpression of PSPTO_4229 resulted in visible difference in the Congo Red assay (Figure 4D). We attempted to identify protein residues or domains necessary for its activity by screening a plasmid expression library for PSPTO_4229 mutants that no longer inhibit motility. We isolated 40 individual PSPTO_4229 mutant expression constructs that failed to inhibit motility when overexpressed and found 30 mutants with frame-shift or nonsense mutations that are predicted to encode truncated proteins. We found 10 mutants predicted to encode full-length protein with between 2 and 6 single amino acid substitutions each and 3 nonsense mutants predicted to encode more than the first 190 amino acids, among which was a mutant that fully preserved the wild-type sequence until amino acid 193 (Supplementary Figure S2). These results suggest that the C-terminal 80 amino acids contain sequences necessary for structure or function of the PSPTO_4229 protein. We also used the ScanNet tool (Tubiana et al., 2022) to scan PSPTO_4229 and found amino acids 84 and 85 as having a high probability of participating in protein–protein interactions, which was close to a mutation hotspot identified in our fail-to-inhibit mutant screen (Figures 5A,B). We cloned a PSPTO_4229 derivative with R84A R85A amino acid substitutions to test its impact on motility. We found replacing these animo acids eliminated motility interference from PSPTO_4229 overexpression (Figures 5C,D).

## Discussion

Bacterial motility regulation is a complex system that evolved to account for specific ecological needs. Here we attempted to confirm the set of genes involved with swimming motility, especially those that had only previously been predicted by orthology, and to identify novel motility regulators in the genome of plant pathogenic bacterium strain *Pst* DC3000. A previous study used a similar *in vitro* evolution approach to identify motility regulators and structural genes in *E. coli* (Girgis et al., 2007) and we built on that idea by combining the random barcoded TnSeq method with *in vitro* selection experiments. We found amino acid biosynthesis was under the strongest selection across all media conditions used in these experiments. Additionally, we identified motility as the function under strongest selection only in the experiments using swimming media. Our data provide empirical evidence for 34 flagellar gene annotations in *Pst* DC3000 and suggest that annotated flagellar motor proteins MotA (PSPTO_4953) and MotB (PSPTO_4952) are antagonistic to swimming motility. We also applied a selection against motility, which allowed us to contrast fitness scores of motility genes in both selection series. This identified known chemotaxis genes *cheAYVRW* and multiple MCPs (Supplementary Table S4), which provides validation for this approach and gives confidence that other genes with similar fitness profiles also contribute to swimming motility. We also identified PSPTO_1042 (*chrR*) and PSPTO_4229, two novel motility genes each with strong motility-altering phenotypes. We used the motility inhibition phenotype from PSPTO_4229 overexpression to screen for functional residues on the hypothetical protein and identified a double arginine residue that is predicted to participate in protein–protein interactions as necessary for this phenotype. Together these results provide a global perspective on regulatory and structural genes controlling flagellar motility in *Pst* DC3000.

Fitness benefits from dispersal are balanced against the energetic expenditure to optimize fitness (Custer et al., 2022). This balance could potentially explain why the fitness scores of motility genes increased in the later passages (Figure 3B).

A bacterial flagellum is a sophisticated transmembrane structure with a motor anchored in the cell wall that drives rotation of the extracellular flagellar filament, which functions like a propellor to push the cell forward (Berg and Anderson, 1973; Doyle et al., 2004; Bouteiller et al., 2021). Flagellar activity and its resulting cellular movement is organized by chemotaxis to control direction in response to chemical gradients in the environment (Sampedro et al., 2015). Producing and operating flagella is also extremely energetically costly, so expression of flagellar genes is carefully managed to provide for the correct functions according to the conditions and to coordinate proper assembly of each flagellum (Dasgupta et al., 2003; Bouteiller et al., 2021). Our results indeed showed 34 *Pst* DC3000 orthologues of the 47 flagellar motility genes in *P. aeruginosa* contributed to growth in soft agar swimming media. Based on this, we propose that *Pst* DC3000 shares a similar 4-tier hierarchy regulating flagellar gene expression but lacks *fliHJ* and *fleL* which are present in *P. aeruginosa* (Figure 2). Our data are also consistent with the previous notion that only *che2* locus genes (and not the *che1* or *che3* loci) are critical for swimming motility in *Pst* DC3000 (Clarke et al., 2016; Munar-Palmer et al., 2024). Notably, we found only one methyl-accepting chemotaxis protein (MCP), PSPTO_0117, among the total 47 MCPs to have a strong fitness phenotype in the motile selection series. This suggests *Pst* DC3000 genome has no redundancy for this MCP and the sensory function provided by PSPTO_0117 is indispensable for growth and expansion in swimming agar based on MOPs minimal medium.

A previous study using *in vitro* evolution to identify motility genes in *E. coli* only used a non-motile selection series (Girgis et al., 2007). After 5 passages, this selection produced a population in which 96% of the individuals had impaired motility and identified 52 out of 54 known motility genes in *E. coli* (Girgis et al., 2007). In contrast, we only reached a rate of about 50% impaired motility after 7 passages. This could potentially be due to the relatively strong selection against loss of motility in minimal medium compared to tryptone medium used by Girgis and coworkers (Girgis et al., 2007), as cells with impaired motility must compete for a smaller pool of nutrients in the minimal medium.

Chemotaxis genes were associated with drastically different fitness scores between the motile and non-motile selection series. These results suggest fitness difference between the motile and non-motile selection series can be used to predict genes that contribute to chemotaxis. However, we do not yet understand why this comparison failed to identify flagellar genes in class I to IV (Supplementary Table S4) (Dasgupta et al., 2003). The absence of fitness score changes from these flagellar regulatory and structural genes suggests the structure of flagella remains beneficial for growth even under selection favoring non-motile strains.

We were surprised to find PSPTO_4953 (*motA-2*) and PSPTO_4952 (*motB*) were deleterious for growth in the motile selection series, which is in stark contrast to the majority of flagella-related genes that contributed to growth in the soft agar swimming medium. This result led to the intriguing hypothesis that wild-type *motAB* functions to inhibit swimming motility in *Pst* DC3000. These genes encode the flagellar stator, an essential structural component for the flagellum to generate torque and facilitate rotation (Doyle et al., 2004; Kanda et al., 2011; Kuchma et al., 2015; Pfeifer et al., 2022). In other *Pseudomonas* species, it is common to have two pairs of flagella stator genes and switching between MotAB to MotCD can favor swimming motility in liquid or semisolid environment, respectively (Doyle et al., 2004; Kanda et al., 2011; Kuchma et al., 2015; Pfeifer et al., 2022). For example, in *P. aeruginosa* null mutations in *motAB* lead to increased swarming motility and only slightly reduced swimming motility (Doyle et al., 2004), while deletion of *motCD* results in loss of swimming in semisolid agar (Kanda et al., 2011; Pfeifer et al., 2022). *Pst* DC3000 has another copy of *motA* (PSPTO_1984) and *motB* (PSPTO_1985) which have been referred to as *motCD* but their annotations have not been updated as such (Toutain et al., 2005). Our data provided more experimental evidence in favor of this hypothesis because the fitness score profile of PSPTO_1984 resembles the ones observed for the *che2 locus* genes and contrasts drastically with the fitness scores for PSPTO_4952 (*motB*) (Supplementary Table S3). This strongly implies functional differences between the two stator pairs in *Pst* DC3000.

We were also surprised to find that *hrpL* and two of its regulator genes *hrpRS* were deleterious for growth in both motile and non-motile selection series (Figure 3C; Supplementary Table S3). The *hrp* genes encode the crucial regulation and structural components of type 3 secretion system (T3SS) in *Pst* DC3000 (Collmer et al., 2000). Activation of *hrp* gene expression and thus T3SS usually requires low pH (~ 5.5) as well as plant-mimicking nutrient environment (Rahme et al., 1992; van Dijk et al., 1999; Zichu Yang et al., 2024). The high fitness scores of *hrp* genes in the motile and non-motile selections indicate these genes were expressed in these conditions and that their expression slows growth, despite that the MOPs minimal media we used has pH range 6.5 ~ 7.9. This suggests 0.2% fructose, acidification from metabolic activity, or the osmolarity of this media may suffice to activate *hrp* gene expression. High energy expenditure of activated T3SS is most likely the reason for relatively slow growth of the strains with the wild type *hrp* gene.

PSPTO_0406 (*dipA*) displayed the largest difference when comparing the fitness scores between the motile and non-motile selection series. Deletion of *dipA* in *P. aeruginosa* can abolish swimming motility in semisolid agar (Mattingly et al., 2018) and asymmetric partition of DipA protein at cell division resulted in significant motility difference in daughter cells (Kulasekara et al., 2013). Surprisingly, the phenotype of our *dipA* deletion was not as drastic. This suggests *dipA* is not essential for swimming in *Pst* DC3000 but is likely a constitutively expressed PDE that modulates motility. Overexpression of *dipA* only partially restored swimming motility to the *dipA* deletion mutant, suggesting wild-type swimming function might require a specific range of *dipA* expression level. In contrast, PSPTO_1042 (*chrR*) showed robust complementation when expressed on a plasmid. The implication of PSPTO_1042 contributing to swimming motility is that upon PSPTO_1043 activation by reactive oxygen species like singlet oxygen (Butcher et al., 2017), cells will reduce flagellar motility as part of the stress response. This contrasts with another example where environmental stresses (including oxidative stress and antibiotic stress) activate bacterial motility (Jones and Elliot, 2017). *P. syringae* encounters oxidative stress on leaf surfaces as well as during activation of plant immunity, with the latter being strong reactive oxygen species burst (Yu et al., 2017; Helmann et al., 2019). Suppression of flagellar gene expression in plants may support full virulence by allowing upregulation of T3SS expression, as the two systems are known to be inversely regulated (Schreiber and Desveaux, 2011; Diepold and Armitage, 2015; Markel et al., 2016). Whether this regulation is relevant in host-pathogen interactions is still unclear and requires further investigation.

Our TnSeq analysis identified PSPTO_4229 as a potentially new regulator of flagellar motility. However, many questions remain unanswered with this hypothetical protein. Despite having fitness scores in both motile and non-motile selection series that were similar to well-known chemotaxis genes, deleting PSPTO_4229 only slightly impaired swimming motility while overexpression abolished motility all together. We found that arginine residues at 84 and 85 were necessary for motility suppression. These residues are predicted to facilitate protein interactions; however, we do not yet have results suggesting the identity of an interaction partner. The similarity between PSPTO_4229 expression phenotypes and the deletion phenotypes of *cheY* suggests PSPTO_4229 might bind and fully interrupt chemotaxis or some other flagellar functions.

Twelve other genes clustered with *motAB* based on their fitness scores profiles (Table 3) that may also make important contributions to motility. PSPTO_0116 is a LysR family transcriptional regulator that is also located immediately upstream of PSPTO_0117, the only methyl-accepting chemotaxis receptor protein identified in the motile selection series. PSPTO_1732 is a hypothetical protein immediately upstream of and likely in the same operon as *bolA*, a transcription factor that positively regulates biofilm formation and repress motility (Dressaire et al., 2015). PSPTO_3229 and PSPTO_3230 are putative homologs of *cdiB* and *cdiA* in the contact-dependent inhibition system of *Pst* DC3000, respectively (Mercy et al., 2016). In *E. coli*, CdiA promotes intercellular adhesion and in turn biofilm formation with the feature of kin discrimination (Ruhe et al., 2015). PSPTO_3890 encodes an isomerase that is predicted as to function as an effector in host-pathogen interaction with *Arabidopsis thaliana,* with its function yet to be characterized (Sahu et al., 2014). PSPTO_5482 encodes a *cheY*-like protein and is located between the phosphate-activated two component system *phoB*/*R* and phosphate transporter protein *phoU*. PhoB regulates swarming motility in *P. aeruginosa* through quorum sensing regulator RhlR (Blus-Kadosh et al., 2013). All the above genes have apparent and plausible connections to regulation of *Pst* DC3000 swimming motility or biofilm formation, making them important targets for future investigations.

## Data Availability

The datasets presented in this study can be found in online repositories. The names of the repository/repositories and accession number(s) can be found in the article/Supplementary material.

## Footnotes

^1^ https://bitbucket.org/berkeleylab/feba/

## References

[ref1] Baltrus D. A. Dougherty K. Diaz B. Murillo R. (2018). Evolutionary plasticity of AmrZ regulation in *Pseudomonas*. mSphere 3:e00132-18. doi: 10.1128/mSphere.00132-18, PMID: 29669886 PMC5907648

[ref2] Bao Z. Wei H.-L. Ma X. Swingle B. (2020). *Pseudomonas syringae* AlgU downregulates Flagellin gene expression, helping evade plant immunity. J. Bacteriol. 202:e00418-19. doi: 10.1128/JB.00418-19, PMID: 31740494 PMC6989796

[ref3] Berg H. C. Anderson R. A. (1973). Bacteria swim by rotating their flagellar filaments. Nature 245, 380–382. doi: 10.1038/245380a0, PMID: 4593496

[ref4] Bertani G. (1951). Studies on lysogenesis. I. The mode of phage liberation by lysogenic *Escherichia coli*. J. Bacteriol. 62, 293–300. doi: 10.1128/jb.62.3.293-300.1951, PMID: 14888646 PMC386127

[ref5] Blus-Kadosh I. Zilka A. Yerushalmi G. Banin E. (2013). The effect of pstS and phoB on quorum sensing and swarming motility in *Pseudomonas aeruginosa*. PLoS One 8:e74444. doi: 10.1371/journal.pone.0074444, PMID: 24023943 PMC3762822

[ref6] Bordi C. Lamy M.-C. Ventre I. Termine E. Hachani A. Fillet S.. (2010). Regulatory RNAs and the HptB/RetS signalling pathways fine-tune *Pseudomonas aeruginosa* pathogenesis. Mol. Microbiol. 76, 1427–1443. doi: 10.1111/j.1365-2958.2010.07146.x, PMID: 20398205 PMC2904497

[ref7] Bouteiller M. Dupont C. Bourigault Y. Latour X. Barbey C. Konto-Ghiorghi Y.. (2021). Pseudomonas flagella: generalities and specificities. Int. J. Mol. Sci. 22:3337. doi: 10.3390/ijms22073337, PMID: 33805191 PMC8036289

[ref8] Buell C. R. Joardar V. Lindeberg M. Selengut J. Paulsen I. T. Gwinn M. L.. (2003). The complete genome sequence of the Arabidopsis and tomato pathogen *Pseudomonas syringae* pv. Tomato DC3000. Proc. Natl. Acad. Sci. USA 100, 10181–10186. doi: 10.1073/pnas.1731982100, PMID: 12928499 PMC193536

[ref9] Butcher B. G. Bao Z. Wilson J. Stodghill P. Swingle B. Filiatrault M.. (2017). The ECF sigma factor, PSPTO_1043, in *Pseudomonas syringae* pv. Tomato DC3000 is induced by oxidative stress and regulates genes involved in oxidative stress response. PLoS One 12:e0180340. doi: 10.1371/journal.pone.0180340, PMID: 28700608 PMC5507510

[ref10] Cassidy C. K. Himes B. A. Sun D. Ma J. Zhao G. Parkinson J. S.. (2020). Structure and dynamics of the *E. coli* chemotaxis core signaling complex by cryo-electron tomography and molecular simulations. Commun. Biol. 3, 24–10. doi: 10.1038/s42003-019-0748-0, PMID: 31925330 PMC6954272

[ref11] Cerna-Vargas J. P. Santamaría-Hernando S. Matilla M. A. Rodríguez-Herva J. J. Daddaoua A. Rodríguez-Palenzuela P.. (2019). Chemoperception of specific amino acids controls Phytopathogenicity in *Pseudomonas syringae* pv. tomato. mBio 10, e01868–e01819. doi: 10.1128/mBio.01868-19, PMID: 31575767 PMC6775455

[ref12] Choi K.-H. Kumar A. Schweizer H. P. (2006). A 10-min method for preparation of highly electrocompetent *Pseudomonas aeruginosa* cells: application for DNA fragment transfer between chromosomes and plasmid transformation. J. Microbiol. Methods 64, 391–397. doi: 10.1016/j.mimet.2005.06.001, PMID: 15987659

[ref13] Christen M. Kulasekara H. D. Christen B. Kulasekara B. R. Hoffman L. R. Miller S. I. (2010). Asymmetrical distribution of the second messenger c-di-GMP upon bacterial cell division. Science 328, 1295–1297. doi: 10.1126/science.1188658, PMID: 20522779 PMC3906730

[ref14] Clarke C. R. Hayes B. W. Runde B. J. Markel E. Swingle B. M. Vinatzer B. A. (2016). Comparative genomics of *Pseudomonas syringae* pathovar tomato reveals novel chemotaxis pathways associated with motility and plant pathogenicity. PeerJ 4:e2570. doi: 10.7717/peerj.2570, PMID: 27812402 PMC5088630

[ref15] Colin R. Ni B. Laganenka L. Sourjik V. (2021). Multiple functions of flagellar motility and chemotaxis in bacterial physiology. FEMS Microbiol. Rev. 45:fuab038. doi: 10.1093/femsre/fuab038, PMID: 34227665 PMC8632791

[ref16] Collmer A. Badel J. L. Charkowski A. O. Deng W.-L. Fouts D. E. Ramos A. R.. (2000). *Pseudomonas syringae* Hrp type III secretion system and effector proteins. Proc. Natl. Acad. Sci. USA 97, 8770–8777. doi: 10.1073/pnas.97.16.8770, PMID: 10922033 PMC34010

[ref17] Custer G. F. Bresciani L. Dini-Andreote F. (2022). Ecological and evolutionary implications of microbial dispersal. Front. Microbiol. 13:855859. doi: 10.3389/fmicb.2022.855859, PMID: 35464980 PMC9019484

[ref18] Dasgupta N. Wolfgang M. C. Goodman A. L. Arora S. K. Jyot J. Lory S.. (2003). A four-tiered transcriptional regulatory circuit controls flagellar biogenesis in *Pseudomonas aeruginosa*. Mol. Microbiol. 50, 809–824. doi: 10.1046/j.1365-2958.2003.03740.x, PMID: 14617143

[ref19] Diepold A. Armitage J. P. (2015). Type III secretion systems: the bacterial flagellum and the injectisome. Philos. Trans. R. Soc. Lond. Ser. B Biol. Sci. 370:20150020. doi: 10.1098/rstb.2015.0020, PMID: 26370933 PMC4632597

[ref20] Doyle T. B. Hawkins A. C. McCarter L. L. (2004). The complex flagellar torque generator of *Pseudomonas aeruginosa*. J. Bacteriol. 186, 6341–6350. doi: 10.1128/JB.186.19.6341-6350.2004, PMID: 15375113 PMC516612

[ref21] Dressaire C. Moreira R. N. Barahona S. Alves de Matos A. P. Arraiano C. M. (2015). BolA is a transcriptional switch that turns off motility and turns on biofilm development. MBio 6, e02352–e02314. doi: 10.1128/mBio.02352-14, PMID: 25691594 PMC4337573

[ref22] Girgis H. S. Liu Y. Ryu W. S. Tavazoie S. (2007). A comprehensive genetic characterization of bacterial motility. PLoS Genet. 3, 1644–1660. doi: 10.1371/journal.pgen.0030154, PMID: 17941710 PMC1976333

[ref23] Helmann T. C. Deutschbauer A. M. Lindow S. E. (2019). Genome-wide identification of *Pseudomonas syringae* genes required for fitness during colonization of the leaf surface and apoplast. PNAS 116, 18900–18910. doi: 10.1073/pnas.1908858116, PMID: 31484768 PMC6754560

[ref24] Jones S. E. Elliot M. A. (2017). Streptomyces exploration: competition, volatile communication and new bacterial Behaviours. Trends Microbiol. 25, 522–531. doi: 10.1016/j.tim.2017.02.001, PMID: 28245952

[ref25] Kanda E. Tatsuta T. Suzuki T. Taguchi F. Naito K. Inagaki Y.. (2011). Two flagellar stators and their roles in motility and virulence in *Pseudomonas syringae* pv. Tabaci 6605. Mol. Gen. Genomics. 285, 163–174. doi: 10.1007/s00438-010-0594-8, PMID: 21165649

[ref26] Khan F. Jeong G.-J. Tabassum N. Kim Y.-M. (2023). Functional diversity of c-di-GMP receptors in prokaryotic and eukaryotic systems. Cell Commun. Signal. 21:259. doi: 10.1186/s12964-023-01263-5, PMID: 37749602 PMC10519070

[ref27] King E. O. Ward M. K. Raney D. E. (1954). Two simple media for the demonstration of pyocyanin and fluorescin. J. Lab. Clin. Med. 44, 301–307, PMID: 13184240

[ref28] Kreiling V. Thormann K. M. (2023). Polarity of c-di-GMP synthesis and degradation. microLife 4:uqad014. doi: 10.1093/femsml/uqad014, PMID: 37251513 PMC10212136

[ref29] Kuchma S. L. Delalez N. J. Filkins L. M. Snavely E. A. Armitage J. P. O’Toole G. A. (2015). Cyclic Di-GMP-mediated repression of swarming motility by *Pseudomonas aeruginosa* PA14 requires the MotAB Stator. J. Bacteriol. 197, 420–430. doi: 10.1128/JB.02130-14, PMID: 25349157 PMC4285984

[ref30] Kulasekara B. R. Kamischke C. Kulasekara H. D. Christen M. Wiggins P. A. Miller S. I. (2013). C-di-GMP heterogeneity is generated by the chemotaxis machinery to regulate flagellar motility. eLife 2:e01402. doi: 10.7554/eLife.01402, PMID: 24347546 PMC3861689

[ref31] Kvitko B. H. Collmer A. (2011). Construction of *Pseudomonas syringae* pv. Tomato DC3000 mutant and polymutant strains. Methods Mol. Biol. 712, 109–128. doi: 10.1007/978-1-61737-998-7_10, PMID: 21359804

[ref32] Lee H.-M. Liao C.-T. Chiang Y.-C. Chang Y.-Y. Yeh Y.-T. Du S.-C.. (2016). Characterization of genes encoding proteins containing HD-related output domain in *Xanthomonas campestris* pv. Campestris. Antonie Van Leeuwenhoek 109, 509–522. doi: 10.1007/s10482-016-0656-y, PMID: 26821378

[ref33] Limoli D. H. Warren E. A. Yarrington K. D. Donegan N. P. Cheung A. L. O’Toole G. (2019). Interspecies interactions induce exploratory motility in *Pseudomonas aeruginosa*. eLife 8:e47365. doi: 10.7554/eLife.47365, PMID: 31713513 PMC6910820

[ref34] Markel E. Stodghill P. Bao Z. Myers C. R. Swingle B. (2016). AlgU controls expression of virulence genes in *Pseudomonas syringae* pv. Tomato DC3000. J. Bacteriol. 198, 2330–2344. doi: 10.1128/JB.00276-16, PMID: 27325679 PMC4984547

[ref35] Matsuyama B. Y. Krasteva P. V. Baraquet C. Harwood C. S. Sondermann H. Navarro M. V. A. S. (2016). Mechanistic insights into c-di-GMP–dependent control of the biofilm regulator FleQ from *Pseudomonas aeruginosa*. PNAS 113, E209–E218. doi: 10.1073/pnas.1523148113, PMID: 26712005 PMC4720306

[ref36] Mattingly A. E. Kamatkar N. G. Morales-Soto N. Borlee B. R. Shrout J. D. (2018). Multiple environmental factors influence the importance of the phosphodiesterase DipA upon *Pseudomonas aeruginosa* swarming. Appl. Environ. Microbiol. 84, e02847–e02817. doi: 10.1128/AEM.02847-17, PMID: 29427430 PMC5861829

[ref37] Mercy C. Ize B. Salcedo S. P. de Bentzmann S. Bigot S. (2016). Functional characterization of Pseudomonas contact dependent growth inhibition (CDI) systems. PLoS One 11:e0147435. doi: 10.1371/journal.pone.0147435, PMID: 26808644 PMC4725963

[ref38] Munar-Palmer M. Santamaría-Hernando S. Liedtke J. Ortega D. R. López-Torrejón G. Rodríguez-Herva J. J.. (2024). Chemosensory systems interact to shape relevant traits for bacterial plant pathogenesis. MBio 15:e0087124. doi: 10.1128/mbio.00871-24, PMID: 38899869 PMC11253619

[ref39] Neidhardt F. C. Bloch P. L. Smith D. F. (1974). Culture Medium for Enterobacteria. J. Bacteriol. 119, 736–747. doi: 10.1128/jb.119.3.736-747.1974, PMID: 4604283 PMC245675

[ref40] Nogales J. Vargas P. Farias G. A. Olmedilla A. Sanjuán J. Gallegos M.-T. (2015). FleQ coordinates flagellum-dependent and -independent motilities in *Pseudomonas syringae* pv. Tomato DC3000. Appl. Environ. Microbiol. 81, 7533–7545. doi: 10.1128/AEM.01798-15, PMID: 26296726 PMC4592877

[ref41] Pérez-Mendoza D. Felipe A. Ferreiro M. D. Sanjuán J. Gallegos M. T. (2019). AmrZ and FleQ co-regulate cellulose production in *Pseudomonas syringae* pv. Tomato DC3000. Front. Microbiol. 10:746. doi: 10.3389/fmicb.2019.00746, PMID: 31057500 PMC6478803

[ref42] Pfeifer V. Beier S. Alirezaeizanjani Z. Beta C. (2022). Role of the two flagellar stators in swimming motility of *Pseudomonas putida*. MBio 13:e0218222. doi: 10.1128/mbio.02182-22, PMID: 36409076 PMC9765564

[ref43] Price M. N. Wetmore K. M. Waters R. J. Callaghan M. Ray J. Liu H.. (2018). Mutant phenotypes for thousands of bacterial genes of unknown function. Nature 557, 503–509. doi: 10.1038/s41586-018-0124-0, PMID: 29769716

[ref44] Rahme L. G. Mindrinos M. N. Panopoulos N. J. (1992). Plant and environmental sensory signals control the expression of hrp genes in *Pseudomonas syringae* pv. Phaseolicola. J. Bacteriol. 174, 3499–3507. doi: 10.1128/jb.174.11.3499-3507.1992, PMID: 1592805 PMC206034

[ref45] Ruhe Z. C. Townsley L. Wallace A. B. King A. der Woude M. W. V. Low D. A.. (2015). CdiA promotes receptor-independent intercellular adhesion. Mol. Microbiol. 98, 175–192. doi: 10.1111/mmi.13114, PMID: 26135212 PMC4694591

[ref46] Rybtke M. T. Borlee B. R. Murakami K. Irie Y. Hentzer M. Nielsen T. E.. (2012). Fluorescence-based reporter for gauging cyclic Di-GMP levels in *Pseudomonas aeruginosa*. Appl. Environ. Microbiol. 78, 5060–5069. doi: 10.1128/AEM.00414-12, PMID: 22582064 PMC3416407

[ref47] Sahu S. S. Weirick T. Kaundal R. (2014). Predicting genome-scale Arabidopsis-*Pseudomonas syringae* interactome using domain and interolog-based approaches. BMC Bioinformatics 15:S13. doi: 10.1186/1471-2105-15-S11-S13, PMID: 25350354 PMC4251041

[ref48] Sampedro I. Parales R. E. Krell T. Hill J. E. (2015). Pseudomonas chemotaxis. FEMS Microbiol. Rev. 39, 17–46. doi: 10.1111/1574-6976.12081, PMID: 25100612

[ref49] Schreiber K. J. Desveaux D. (2011). AlgW regulates multiple *Pseudomonas syringae* virulence strategies. Mol. Microbiol. 80, 364–377. doi: 10.1111/j.1365-2958.2011.07571.x, PMID: 21306444

[ref50] Swingle B. Thete D. Moll M. Myers C. R. Schneider D. J. Cartinhour S. (2008). Characterization of the PvdS-regulated promoter motif in *Pseudomonas syringae* pv. Tomato DC3000 reveals regulon members and insights regarding PvdS function in other pseudomonads. Mol. Microbiol. 68, 871–889. doi: 10.1111/j.1365-2958.2008.06209.x, PMID: 18363796

[ref52] Toutain C. M. Zegans M. E. O’Toole G. A. (2005). Evidence for two flagellar stators and their role in the motility of *Pseudomonas aeruginosa*. J. Bacteriol. 187, 771–777. doi: 10.1128/JB.187.2.771-777.2005, PMID: 15629949 PMC543560

[ref53] Tubiana J. Schneidman-Duhovny D. Wolfson H. J. (2022). ScanNet: an interpretable geometric deep learning model for structure-based protein binding site prediction. Nat. Methods 19, 730–739. doi: 10.1038/s41592-022-01490-7, PMID: 35637310

[ref54] Usuki G. Ishiga T. Sakata N. Ishiga Y. (2024). Flagellar motility of *Pseudomonas syringae* pv. Actinidiae biovar 3 contributes to bacterial infection through stomata. J. Gen. Plant Pathol. 90, 144–150. doi: 10.1007/s10327-024-01172-6

[ref55] van Dijk K. Fouts D. E. Rehm A. H. Hill A. R. Collmer A. Alfano J. R. (1999). The Avr (effector) proteins HrmA (HopPsyA) and AvrPto are secreted in culture from *Pseudomonas syringae* pathovars via the Hrp (type III) protein secretion system in a temperature- and pH-sensitive manner. J. Bacteriol. 181, 4790–4797. doi: 10.1128/JB.181.16.4790-4797.1999, PMID: 10438746 PMC93963

[ref56] van Opijnen T. Bodi K. L. Camilli A. (2009). Tn-seq: high-throughput parallel sequencing for fitness and genetic interaction studies in microorganisms. Nat. Methods 6, 767–772. doi: 10.1038/nmeth.1377, PMID: 19767758 PMC2957483

[ref57] Wadhwa N. Berg H. C. (2022). Bacterial motility: machinery and mechanisms. Nat. Rev. Microbiol. 20, 161–173. doi: 10.1038/s41579-021-00626-4, PMID: 34548639

[ref58] Wang F. Deng L. Huang F. Wang Z. Lu Q. Xu C. (2020). Flagellar motility is critical for *Salmonella enterica* Serovar typhimurium biofilm development. Front. Microbiol. 11:1695. doi: 10.3389/fmicb.2020.01695, PMID: 33013719 PMC7509047

[ref9001] Wei C. -F. Kvitko B. H. Shimizu R. Crabill E. Alfano J. R. Lin N. -C.. (2007). A Pseudomonas syringae pv. tomato DC3000 mutant lacking the type III effector HopQ1-1 is able to cause disease in the model plant Nicotiana benthamiana. Plant J. 51, 32–46. doi: 10.1111/j.1365-313X.2007.03126.x 17559511

[ref59] Wetmore K. M. Price M. N. Waters R. J. Lamson J. S. He J. Hoover C. A.. (2015). Rapid quantification of mutant fitness in diverse Bacteria by sequencing randomly Bar-coded transposons. mBio 6, e00306–e00315. doi: 10.1128/mBio.00306-15, PMID: 25968644 PMC4436071

[ref60] Yang Z. Wang H. Keebler R. Lovelace A. Chen H.-C. Kvitko B.. (2024). Environmental alkalization suppresses deployment of virulence strategies in *Pseudomonas syringae* pv. Tomato DC3000. J. Bacteriol. 206:e0008624. doi: 10.1128/jb.00086-24, PMID: 39445803 PMC11580431

[ref61] Yarrington K. D. Shendruk T. N. Limoli D. H. (2024). The type IV pilus chemoreceptor PilJ controls chemotaxis of one bacterial species towards another. PLoS Biol. 22:e3002488. doi: 10.1371/journal.pbio.3002488, PMID: 38349934 PMC10896506

[ref62] Yu H. S. Alam M. (1997). An agarose-in-plug bridge method to study chemotaxis in the archaeon *Halobacterium salinarum*. FEMS Microbiol. Lett. 156, 265–269. doi: 10.1111/j.1574-6968.1997.tb12738.x, PMID: 9513275

[ref63] Yu X. Feng B. He P. Shan L. (2017). From Chaos to harmony: responses and signaling upon microbial pattern recognition. Annu. Rev. Phytopathol. 55, 109–137. doi: 10.1146/annurev-phyto-080516-035649, PMID: 28525309 PMC6240913

