# Supplementary material for: Genome-wide identification of novel flagellar motility genes in Pseudomonas syringae pv. tomato DC3000
Source: Front Microbiol. 2025 Jan 28;16:1535114. doi: 10.3389/fmicb.2025.1535114 (PMC11813219; doi:10.3389/fmicb.2025.1535114)
Supplement: Supplementary file 1 [file Supplementary_file_1.zip › Supplemental Figure S1 and Figure S2.docx]

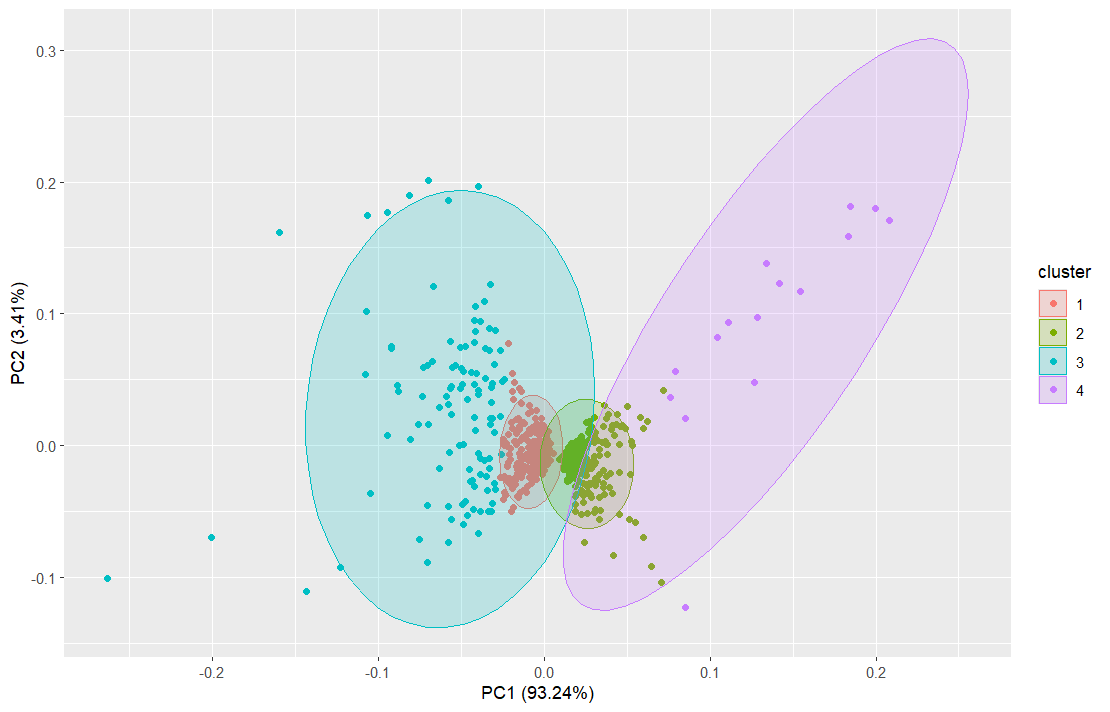
Supplement Figure S1. Principal component analysis (PCA) of genomic per-passage fitness scores difference separates all 600 identified differentially fit genes. Clusters were assigned by K-means analysis with cluster number = 4. Cluster 3 and 4 corresponds with the identified *che* and *mot* group of genes.


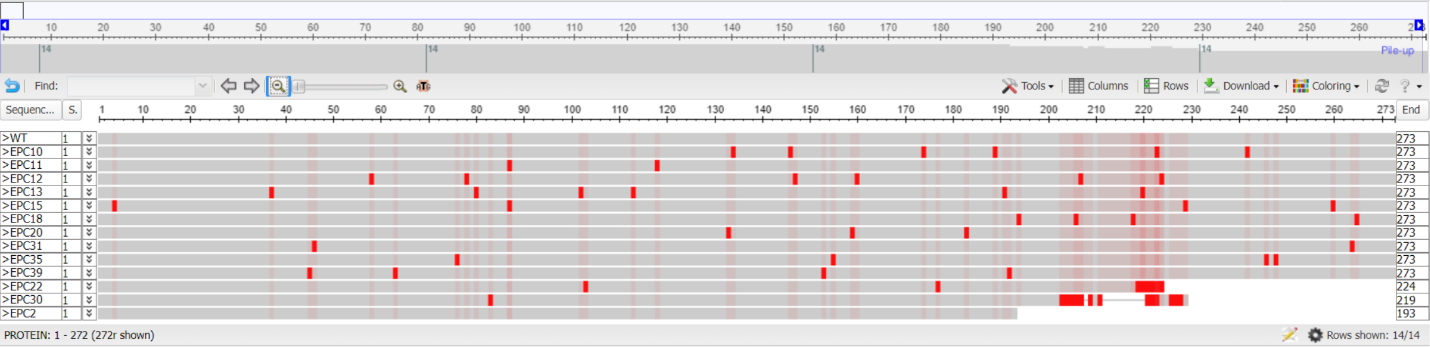


Supplement Figure S2. Amino acid sequence alignment of mutant PSPTO_4229 in overexpression constructs. 13 sequences from mutants that lost motility suppression phenotype when overexpressed preserved more than two-thirds of wild-type PSPTO_4229 amino acid sequences.
